# Evaluation of aging effects on the physical, chemical, and mechanical properties of thermoplastic materials for orthodontic clear aligners: an in vivo study

**DOI:** 10.1186/s12903-025-06966-4

**Published:** 2025-10-08

**Authors:** Fatemeh Arabpour, Hossein Agha Aghili, Maryam Javaheri Mahd, Zahra Arabpour, Soghra Yasaei

**Affiliations:** 1 Department of Orthodontics, School of Dentistry, Arak University of Medical Sciences, Arak, Iran; 2 Department of Orthodontics, School of Dentistry, Shahid Sadoughi University of Medical Sciences, Yazd, Iran; 3 Student Research Committee, Iran University of Medical Sciences, Tehran, Iran

**Keywords:** Clear aligners, Aging, Physical properties, Mechanical properties, Chemical properties

## Abstract

**Background:**

Due to the viscoelasticity of orthodontic thermoplastic materials, the force generated by the orthodontic device for the same deformation will decline over time. Aligners are continually exposed to saliva at a constant body temperature, which can have an adverse effect on the chemical composition of the polymers. More studies are needed to investigate the in vivo aging of other materials, to determine the Best material that could meet treatment needs. We show the comparison of physical, chemical, and mechanical changes of clear aligners before and after 10 days of intraoral usage.

**Methods:**

Twenty-five unused aligners and twenty-five aligners used in the oral environment were first gathered from five patients requiring clear aligners and then subjected to water absorption and solubility, tensile strength, modulus of elasticity, and Chemical properties testing. The Kolmogorov-Smirnov (K-S) test was used to compare the equality of distributions. The Wilcoxon test was used to determine the median difference by time. Data were analyzed in R-4.4.1 using the ggstatsplot package and ggwithinstats function. The results with *p* < 0.05 were considered statistically significant for all tests.

**Results:**

The mean water solubility (Wsl) was zero (before using aligners) and 0.00013 (after using aligners), indicating an insignificant difference between the two time periods (*p* = 0.150). The mean Max Force (N) was 97.105 (before using aligners) and 111.18 (after using aligners), implying an insignificant difference between the two time periods (*p* = 0.640). Similarly, the mean normalized displacement (%) was 123.56 (before using aligners) and 122.08 (after using aligners), indicating an insignificant difference between the two time periods (*p* = 0.290). The chemical composition analysis revealed the hydrolysis of ester bonds to carboxylic acid and alcohol functional groups post-treatment.

**Conclusion:**

The aligners’ mechanical and physical properties are not significantly affected, but their chemical properties can be altered with aging.

## Introduction

Aligner orthodontic therapy has markedly revolutionized dentistry and orthodontics [1]. Research has recently advocated that aligner orthodontic therapy accounts for 30% to 45% of all orthodontic therapies, and there are coincident alterations in the patient’s preferences and awareness of aligner orthodontic therapy [2–4]. Since the materials used in clear aligners should possess superb mechanical properties to ensure the efficacy of orthodontic therapies, the technical data delivered by the suppliers cannot be a reliable source in all cases, and the materials used in clinical applications need to pass strict experimental evaluations under real and diverse conditions. Importantly, aligners are exposed to an aggressive environment in the oral cavity, and this can potentially disturb their properties and adversely affect the efficacy of the aligner orthodontic therapy [5].

Due to the viscoelasticity of these materials, the force generated by the orthodontic device for the same deformation will decline with aging. Such a tension reduction is affected by the material, the oral environment’s temperature, and the intensity of the load affecting a certain part of the aligner [6]. Furthermore, aligners are continually exposed to saliva at a constant body temperature, which can adversely affect the chemical composition of the polymers. Certain polyesters (e.g., polycarbonates and polyamides) may exhibit irreversible hydrolysis that may lead to the eventual degradation of their polymer structure [7]. Furthermore, chemical degradation may trigger the release of elements in the oral cavity and cause poisoning or allergies [8].

According to systematic reviews [9], the effects of aging on aligner materials (except for Invisalign) need to be precisely assessed in vivo to determine the best material that meets the therapy requirements. Therefore, the present research evaluates the changes in the physical, mechanical, and chemical properties of clear aligners following intraoral use.

## Materials and methods

### Study design

Twenty-five unused aligners and twenty-five aligners used in the oral environment were first gathered from five patients requiring clear aligners (five patients were enrolled in the study, and five aligners were retrieved from each patient after 10 days of intraoral use, resulting in a total of 25 used specimens. For each of these used aligners, an identical unused aligner was fabricated in the laboratory using the same digital design and thermoforming process). These lab-fabricated aligners served as the control group. The aligners are made of polyethylene terephthalate glycol. Aligners were randomly selected using random allocation software to choose five patients who required aligner orthodontic therapy with clear aligners. The inclusion criteria were 1) being a patient eligible for aligner orthodontic therapy, 2) signing the consent form to attend the study, and 3) using the clear aligner for 20 ± 2 h a day. The exclusion criteria were 1) unwillingness to attend the research and 2) not using the clear aligner for 20 ± 2 h a day.

After collecting aligners, a copy of them was fabricated by the laboratory to be evaluated as unused aligners. Accordingly, the research groups included 1) the T0 group (i.e., not used aligners) and 2) the T10 group (i.e., aligners used for 10 days in the oral environment). To reduce bias, all samples were coded and tested in random order, with the operator blinded to the group allocation.

### Physical properties

Water absorption (Wsp) and water solubility (Wsl) tests were based on the 2013 standard ISO 20795-2:2013. Aligners were placed in a 40 °C incubator and kept at a constant temperature of 37 ± 1 °C for 24 ± 1 h. They were then weighed on a scale with a readability of 0.0002 gr. The drying-and-weighing cycle was repeated three times until reaching the conditional mass (m1). Next, the samples were immersed in distilled water at a constant temperature of 37 ± 1 °C. After five days, they were removed from the water and then gently wiped to remove visible moisture, and weighed after 60 s of water removal. The samples were re-immersed in water and re-measured every 4 to 5 days until reaching water saturation mass (m2). Afterward, the samples were removed from the water and dried in the incubator to reach a reconstituted mass (m3).

The middle third of the maxillary central incisor teeth (5 × 5) were cut from the aligners to be evaluated in this research. The reason for choosing this part is that it is fairly smooth geometrically and affords more robust results in our testing system compared to the curved part of the aligner.

The procedure for evaluating water absorption and solubility was generally based on the ISO 20795-2:2013 standard. However, two controlled deviations from the ISO protocol were applied. First, according to the standard, specimens are typically required to be 10 × 10 mm² in size. However, in our study, sections were obtained directly from the labial surface of the maxillary central incisor region of clinically used aligners. Due to the anatomical constraints and limited flat surface area of the aligners in this region, it was not feasible to obtain specimens of the standard 10 × 10 mm size without altering the structural integrity or introducing curvature-induced variability. Therefore, smaller specimens measuring 5 × 5 mm² were used, as this region offers the flattest geometry available, providing the most consistent and representative area for testing. This modification ensured methodological practicality without compromising data validity. Second, instead of using a fixed 7-day immersion period, the samples were submerged in distilled water at 37 ± 1 °C and weighed at 4- to 5-day intervals. The immersion time was set to 5 days instead of 7 days according to ISO, based on protocol feasibility and consistency with previous similar studies.

The water absorption (Wsp) capacity of the samples (i.e., five samples from each group) was calculated using the equation below:

$$\:W_{sp}=\frac{m_2-m_3}v\left(\frac{\mu g}{{mm}^3}\right)$$

Where ($\:{m}_{2}$) is the sample’s mass (in $\:\mu g$) after water immersion, ($\:{m}_{3}$) is the sample’s reconstituted mass (in $\:\mu g$), and ($\:v$) is the sample’s volume ($\:{mm}^{3}$). 

Water solubility (Wsl) was calculated from the equation below:

$$\:W_{sl}=\frac{m_1-m_3}v\left(\frac{\mu g}{{mm}^3}\right)$$

Where ($\:{m}_{1}$) is the sample’s mass (in $\:\mu\:g$) before water immersion.

### Mechanical properties

#### Modulus of elasticity

**Fig. 1:**
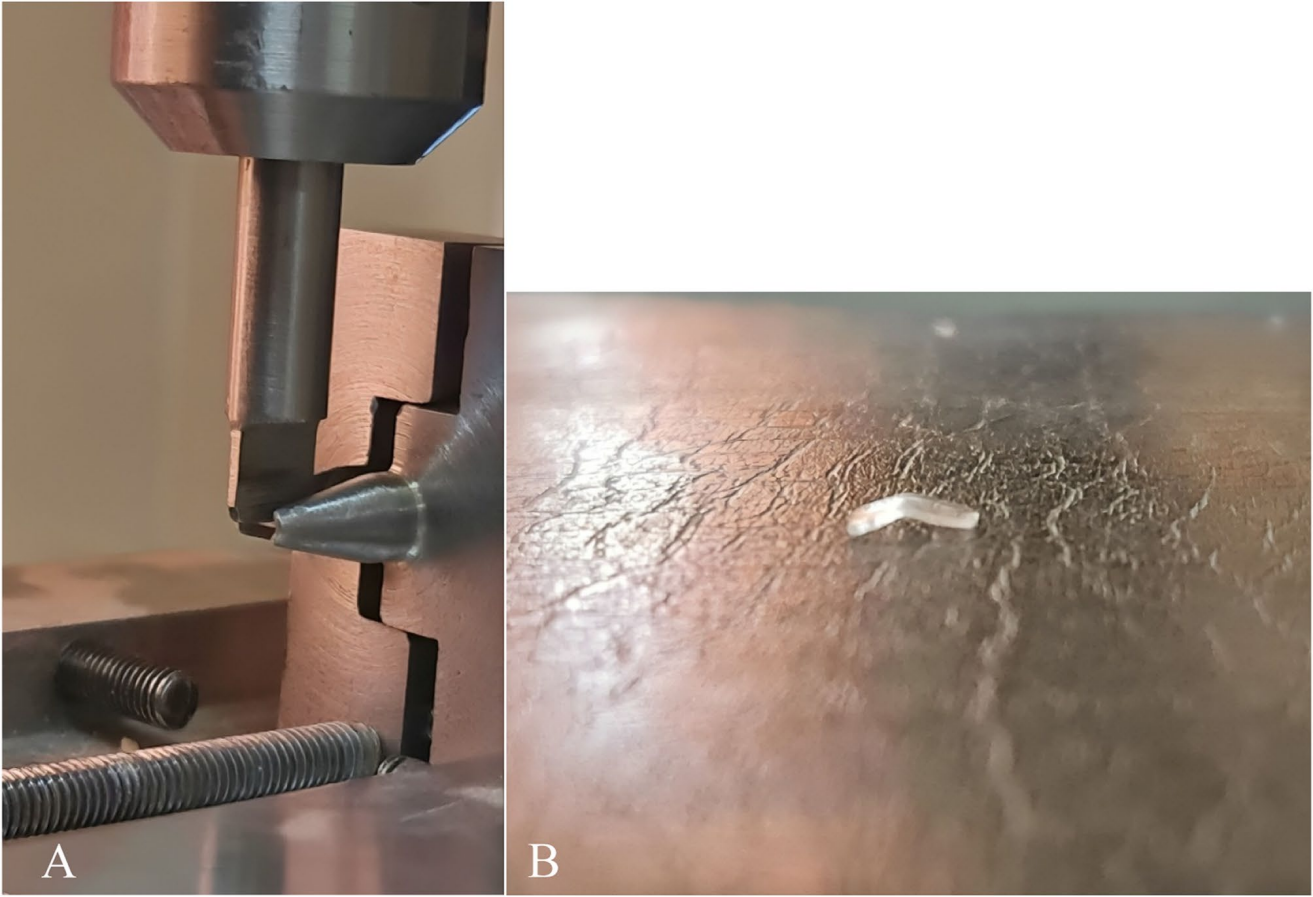
Three-point bending test, **A**: the specimen positioned inside the testometric testing machine, **B**: the specimen after completion of the test

The three-point bending test was performed to measure the modulus of elasticity in both groups. Five samples from each group (each sample: 5 × 2.5 mm) were prepared from the central maxillary tooth surface. The flexure modulus was measured for each sample using a testometric testing machine (Model: DBBMTCL-1000 kg, ROCHADL, ENGLAND). The strain distance of 0.5 mm was adjusted (i.e., from 0.5 to 1.0 mm, based on previous studies) at a speed of 5 mm/min (Fig. 1). The flexure modulus was measured using the equation below:

$$\:E=\frac{(F_2-F_1)I^3}{{4bh}^3(d_2-d_1)}$$

Where ($\:{F}_{1}$) is the maximum force at 0.5 mm deflection, ($\:{F}_{2}$) is the maximum force at 1.0 mm deflection, ($\:{d}_{1}$) is 0.5 mm deflection, ($\:{d}_{2}$) is 1.0 mm deflection, ($\:I$) represents distance, ($\:b$) denotes width. ($\:h$) denotes height, and ($\:E$) is the flexure modulus.

#### Tensile strength

The flexure strength was measured according to Condo et al. [10]. For this, maxillary dental arch aligners were used, and two flat clamp pads were attached to the tension clamp of the testometric device (Model: DBBMTCL-1000 kg, ROCHADL, ENGLAND). Based on the characteristic arch geometry of the aligners, five samples from each group were tested in the C-ring flexure test. The elastic properties of aligners in both groups were identified by subjecting the samples to a load at a speed of 5 mm/min until they reached their elastic limit.

Then, two issues were examined. The force that the aligner withstands before entering the elastic phase. And two similar points from the aligner were measured in millimeter, before and after stretching, to show the aligner’s ability to be stretched, which will be known as normalized displacement.

### Chemical properties

The chemical composition of aligners was identified via attenuated total reflectance Fourier transform infrared (ATR-FTIR) spectroscopy. After cutting, the buccal surface of the samples was precisely investigated.

### Statistical analysis

The sample size for this study was calculated by Using G-Power software and similar study values of Ryu JH et al. [11] the mean of the first group was 0.46 and the second group was 0.16, and the standard deviations were 0.07 and 0.02, considering a type I error of 0.05, a test power of 0.95, and a correlation of 0.20, 4 samples were calculated for each factor. Considering attrition, the final sample size was 5 for each factor.

The information of five patients undergoing aligner orthodontic therapy was collected, and research hypotheses were investigated in a Table and three graphs. The Kolmogorov–Smirnov (K-S) test was performed to compare the equality of distributions. The Wilcoxon test (a non-parametric equivalent to the paired samples t-test) was used to determine the median difference by time. Data were analyzed in R-4.4.1 using the ggstatsplot package and ggwithinstats function. The results with *p* < 0.05 were considered statistically significant for all tests.

Table 1 tabulates the minimum, maximum, mean, standard deviation (SD), and normalized values regarding the physical and mechanical properties of the research samples. The non-parametric test (*p* < 0.05) was used for the Wsl factor, and the parametric test (*p* < 0.05) was used for other factors.

**Table 1:** The physical and mechanical properties of the research samples

Factors	Time	Max : Min	Mean ± SD	*p*-value*
Wsl	Before	0 : 0.00013	0.0000026 ± 0.0006	˂0.001
	After	0 : 0.00013	0.000011 ± 0.0006	˂0.001
Wsp	Before	0.00013 : 0.00013	0.00013 ± 0	-
	After	0.00013 : 0.00013	0.00013 ± 0	-
Max force (N)	Before	130.47 : 72.70	105.97 ± 21.84	0.200
	After	146.20 : 72.25	111.18 ± 26.32	0.200
Normalized displacement (%)	Before	146.20 : 112.72	123.56 ± 11.39	0.200
	After	138.29 : 108.92	122.08 ± 11.38	0.200
Flexure modulus (MPa)	Before	361.47 : 17.77	142.22 ± 148.73	0.127
	After	458.92 : 5.92	122.08 ± 11.38	0.200

*Wsl *Water solubility *Wsp* Water absorption

*Normality investigation by the Kolmogorov–Smirnov (K-S) test

p-value < 0.05 was considered significant

## Results

Of the five patients treated with aligners, five of their aligners were received after 10 days of use, while an unused copy of the same aligners was available, to investigate the effect of aging on physical properties, including water absorption and solubility, mechanical properties, including elastic modulus and tensile strength, and chemical properties of the aligners.

### Water solubility

**Fig. 2:**
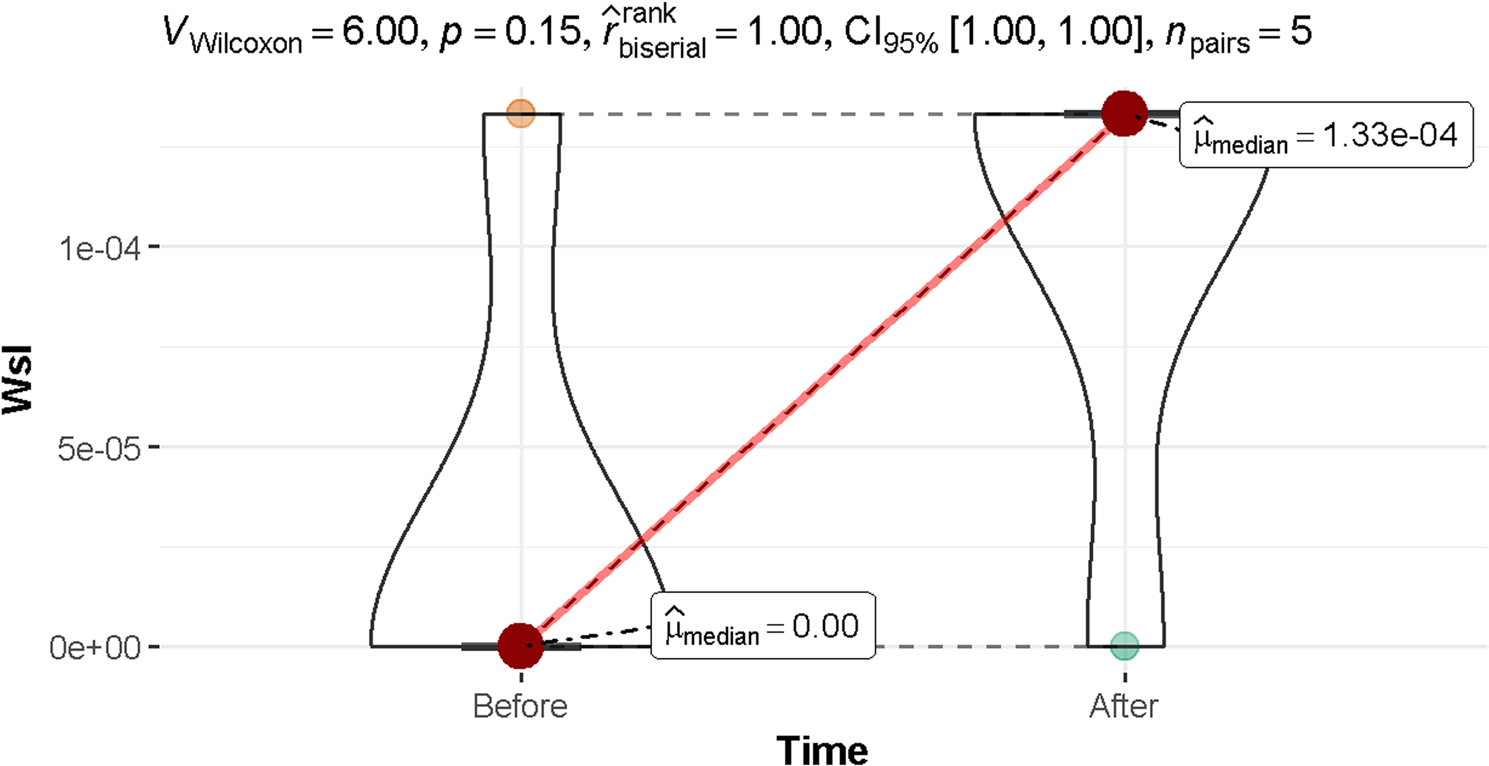
The median Wsl value before and after using aligners p-value < 0.05 was considered significant. CI: confidence interval

As shown in Fig. 2, the median Wsl is zero (before using aligners) and 0.00013 (after using aligners), indicating an insignificant difference between the two time periods (95% confidence interval (CI): 1.00 to 1.00, *p* = 0.150).

### Water absorption

No change in the water absorption of the aligners was observed before and after 10 days of use.

### Tensile strength

In this experiment, two aspects were evaluated: The maximum force that the aligner can withstand before entering the elastic phase (Max Force (N)). The measurement of two corresponding points on the aligner, set at a millimetric distance, to represent the aligner’s tensile capacity as millimetric displacement values (normalized displacement (%)).

**Fig. 3:**
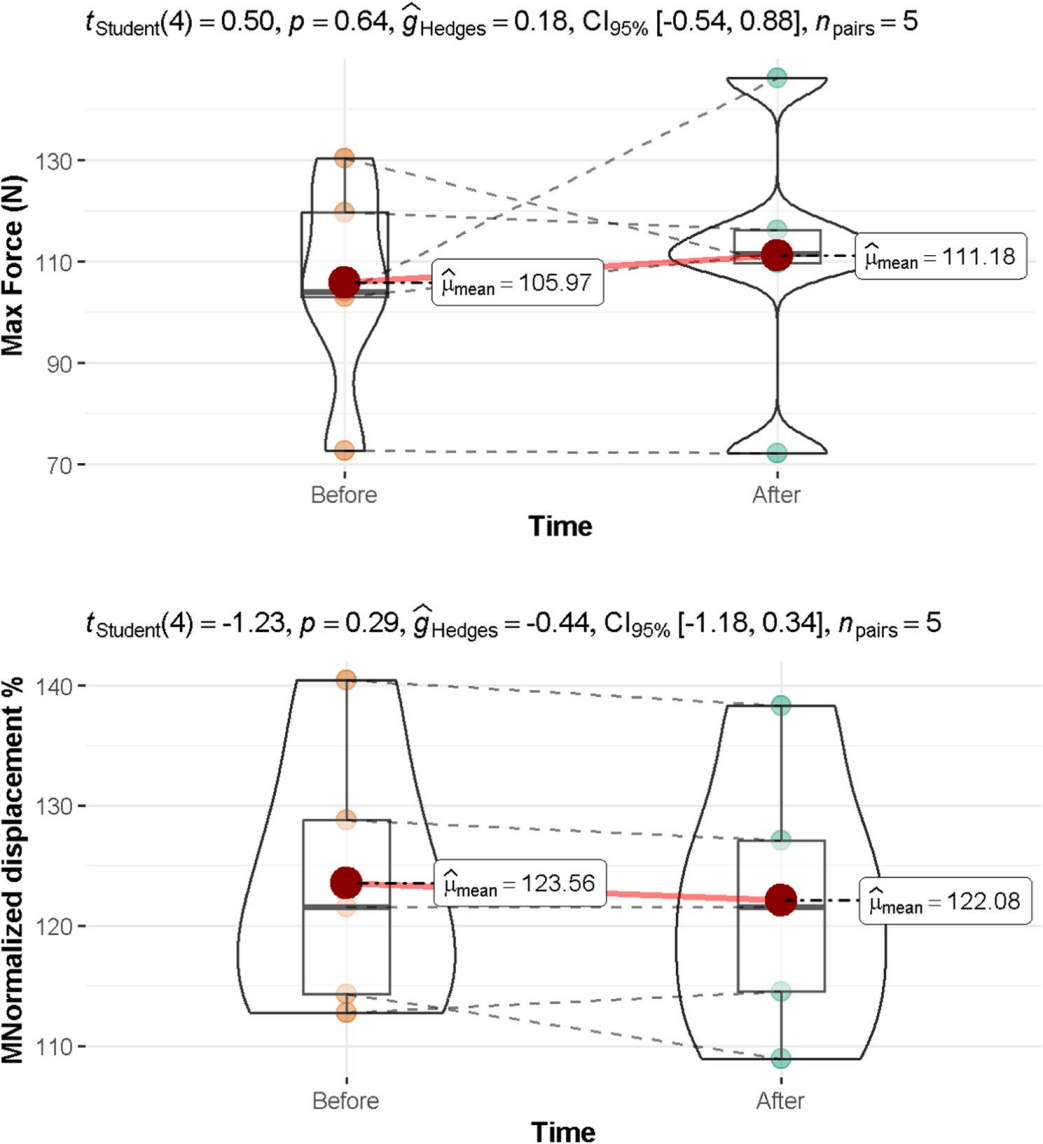
The mean max force (N) and normalized displacement (%) values before and after using aligners p-value < 0.05 was considered significant. CI: confidence interval

As shown in Fig. 3, the mean Max Force (N) is 105.97 (before using aligners) and 111.18 (after using aligners), implying an insignificant difference between the two time periods (95% CI: −0.54 to 0.88, *p* = 0.640). Similarly, the mean normalized displacement (%) is 123.56 (before using aligners) and 122.08 (after using aligners), indicating an insignificant difference between the two time periods (95% CI: −1.18 to 0.34, *p* = 0.290).

### Modulus of elasticity

**Fig. 4:**
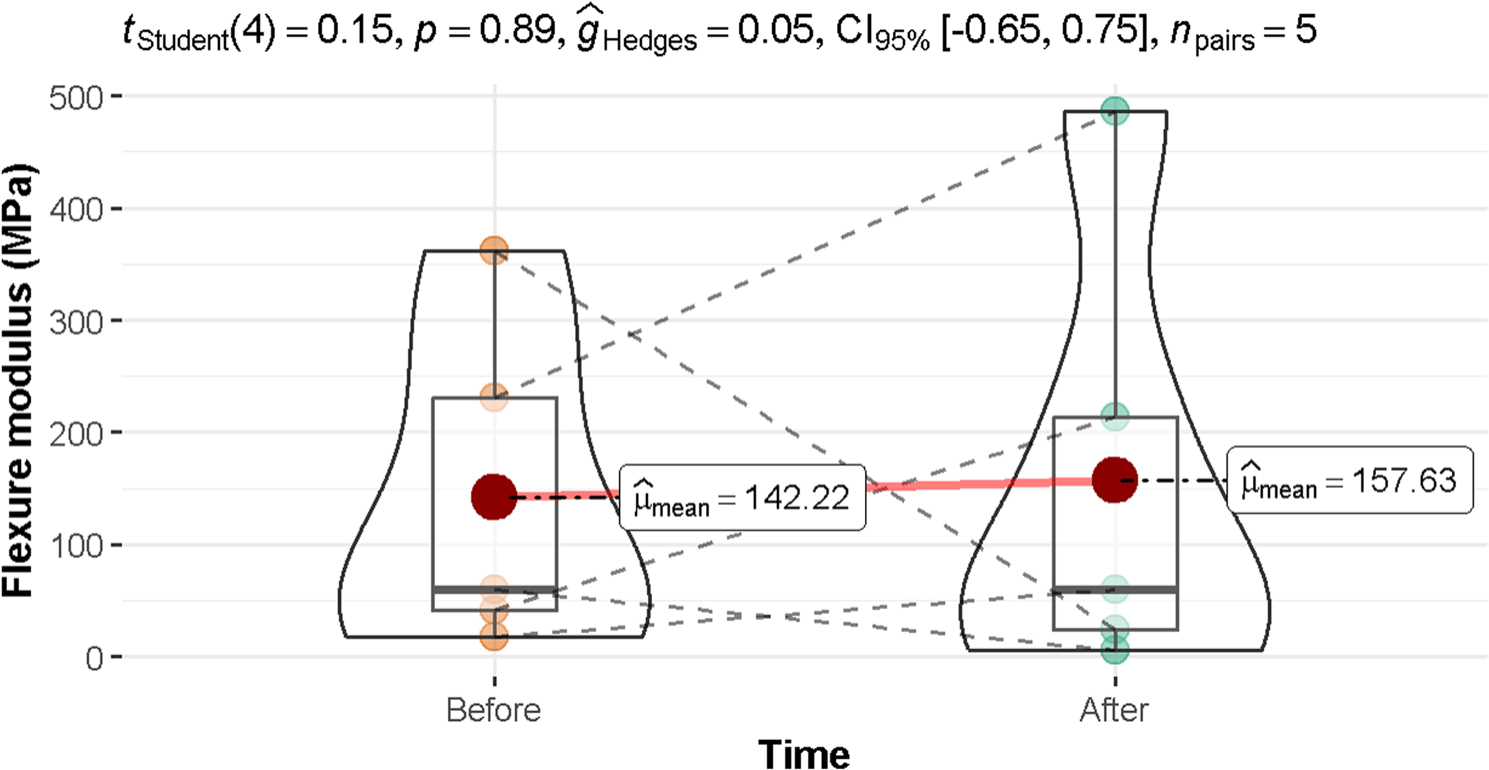
The mean flexure modulus (MPa) before and after using aligners p-value < 0.05 was considered significant. CI: confidence interval

As shown in Fig. 4, the mean flexure modulus (MPa) is 142.22 (before using aligners) and 157.63 (after using aligners), implying an insignificant difference between the two time periods (95% CI: −0.65 to 0.75, *p* = 0.890).

### Chemical properties

The chemical changes before and after using aligners are shown in Table 2.

**Table 2:** Chemical composition of samples according to wave number before and after aligner orthodontic therapy

Sample		Wavenumber (cm^−1^)	Assignments	Group
A	Before	742	C-H (out-of-plane bend)	Aromatics
		1095	C-O	Ester
		1247	C-O	Ester
		1717	3 or 4 small humps	Aromatic
	After	(1220)	C-O	Ester
		(1363)	-CH3 (bend)	Alkanes
		(1720)	3 or 4 small humps	Aromatic
B	Before	724	C-H (out-of-plane bend)	Aromatics
		1096	C-O	Ester
		1248	C-O	Ester
		1717	3 or 4 small humps	Aromatic
	After	(2356)	C-H bonds	Isocyanates
		(3650)	O-H	Carboxylic acids
C	Before	723	C-H (out-of-plane bend)	Aromatics
		1095	C-O	Ester
		1247	C-O	Ester
		1716	3 or 4 small humps	Aromatics
	After	(3512)	O-H	Alcohols
		(3607)	O-H	Carboxylic acids
D	Before	2340	C-H bonds	Isocyanates
		3438	O-H	Alcohols
		3637	O-H	Alcohols
	After	(3460)	O-H	Alcohols
		(3540)	O-H	Alcohols
		(3611)	O-H	Alcohols
		(3819)	O-H	Alcohols
E	Before	2340	C-H bonds	Isocyanates
		2923	C-H bond vibration CH_2_ and CH_3_	Alkanes
	After	(724)	C-H (out-of-plane bend)	Aromatics
		(1095)	C-O	Ester
		(1247)	C-O	Ester
		(1716)	3 or 4 small humps	Aromatics
		(2925)	O-H	Carboxylic acids

**Fig. 5:**
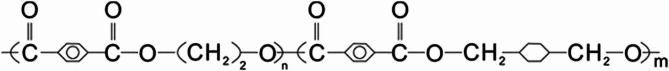
The structure of polyethylene terephthalate glycol

Regarding the structure of polyethylene terephthalate glycol (Fig. 5), ester bonds were hydrolyzed to carboxylic acid and alcohol functional groups after aligner orthodontic therapy, except for sample A, which experienced no significant change.

## Discussion

This research evaluated the mechanical, physical, and chemical properties of fifty used and unused aligner samples in vivo. Previous research has mostly investigated the properties of aligners in vitro and mostly evaluated Invisalign aligners, which are made from polyurethane. This research evaluated aligners made from polyethylene terephthalate glycol in vivo.

Also in most previous investigations, the replacement interval of aligners has been fixed at 14 days, and significant changes were reported over this period. However, in daily practice, the replacement interval is frequently shortened to 10 days after the initial phase of treatment. We therefore selected a 10-day interval to both reflect this widely used clinical approach and to examine whether material changes would still occur in a shorter yet clinically relevant timeframe.

### Physical properties

Based on previous research, water absorption reduces tension in the matrix of thermoplastic materials via plasticization, a phenomenon that triggers intra-chain and/or inter-chain debonds, alters the free volume of polymers, washes soluble components, and enhances the degradation process [12–16]. The transparency and color stability of clear appliances are further affected by water absorption. Importantly, since the appearance of such appliances is paramount, their optical properties are supposed to be preserved [17, 18].

The rate of water absorption in used and unused aligners was trivial, implying an insignificant difference between the two time periods. The water solubility of used aligners was higher than that of unused aligners, but the difference was statistically​​ insignificant. Recent research on Invisalign aligners revealed a gradual increase in water absorption in polyurethane aligners after using them [19]. In another study, polyurethane aligners had a higher rate of water absorption in distilled water compared to the aligners made up of polyethylene terephthalate glycol and polycarbonate materials [20], indicating that the difference is due to the diverse materials employed and the duration of use of the aligner was attributed, so that in the present study, the duration of use of each aligner was 10 days, while previous studies had been 14 days. So we can say that after 10 days of use, the aligner has not lost its properties in this regard.

### Mechanical properties

Although the properties of aligners are influenced after use, the difference between used and unused aligners is insignificant. These mechanical properties of aligners were evaluated regarding the modulus of elasticity and tensile strength.

#### Modulus of elasticity

Using aligners in the oral cavity for 10 days increases their modulus of elasticity, but it was not statistically significant. Ferreira Lira et al. [19] studied Invisalign aligners and found a significant decline in the modulus of elasticity after two weeks of use, indicating that the aligners have become softer. This difference may be due to different times and types of aligners, i.e., polyurethane aligners compared to polyethylene terephthalate glycol aligners. A study by Tamburrino et al. [21] on three different brands of aligners, in vitro stored in artificial saliva, found that the elastic modulus of the Duran and Zendura brands decreased significantly. However, the elastic modulus of the Biolon brand was quite stable, which is similar to the result of this study, which found no significant difference in elastic modulus changes after intraoral use. while, Condo et al. [10] studied two aligner brands and reported structural changes in both brands that enhanced sample hardness and hyperplasticity.

An enhancement in the modulus of elasticity (which was seen in this study, although it was not statistically significant) may be due to the release of matrix plasticizers into the oral environment after use, making the material stiffer. In the clinic, this can be seen as a loss of flexibility and adaptability of the aligner to the teeth and attachments after use by the patient. This change causes tooth movement not to follow the planned movements. Also, a stiffer aligner can affect aligner seating, attachment engagement, or patient comfort.

In another study [22] which compared single-layer and multilayer aligners concluded that thermomechanical aging significantly reduced forces and moments during the first 48 h. Multilayer aligner materials exhibit lower initial forces and moments than single-layer ones, and were less influenced by aging. Which is in contrast of our study on sngle-layer aligner that has no statistical difference after aging in modulus of elasticity that suggest that the forces shouldn’t get al.tered.

Since 10-day intervals of aligner replacement were considered in this study, and no statistically significant change was observed, it can be concluded that the aligner does not lose its flexibility within 10 days and that a change in the stiffness of the aligner is not a reason to replace the aligner within 10 days.

#### Tensile strength

The importance of examining the tensile strength of aligners is that aligners with low tensile strength may break quickly, which can affect proper therapeutic feedback and reduce the efficiency and quality of treatment.

In this test, both the maximum force that the aligner could withstand before it entered the plastic phase and the amount of distance at two similar points on the aligner to indicate how much it could be stretched were evaluated.

In this experiment, two issues were examined:

- 1- The force that the aligner withstands before entering the elastic phase.
- 2- Two similar points from the aligner were measured to show the aligner’s ability to be stretched in millimeters.

The aligners, before use, withstood less force before entering the plastic phase, but more distance was created between two similar points. That means, in the elastic range, with less force, more stretching was done. It could be said that the aligners may have exhibited increased brittleness after use.

Such a test method has only been performed in one other study [10] on two different brands of aligners (LD30N and EX30N), and compared to their study, our samples both withstood more force and moved further apart, indicating that the brand used in this study, which was made of PET-G, performed better in terms of tensile strength.

The current study did not find a statistically significant difference in changes in this material property after 10 days of intraoral use of the aligner, which could mean that the aligner maintains its tensile strength after 10 days, and during this period, we should not witness issues such as breakage in the aligner.

### Chemical properties

Ester bonds were hydrolyzed to carboxylic acid and alcohol functional groups after aligner orthodontic therapy in most of the samples. Water chemically reacts with the polymer matrix during hydrolysis, and this markedly alters the aligner’s structure and properties. Notably, hydrolysis causes irreversible degradation of the polymers. Research reports dimensional alterations in PET-G materials, where water infiltrates into the polymers and alters their structure and surface [23]. Clinically, the results of this study indicate that orthodontic forces lead to polymer wear during orthodontic treatment, which has been confirmed by previous studies [5, 23, 24]. Aligners generate orthodontic forces in the first 24 h. Next, this force follows a plateau trend and remains constant until the end of 14 days of use [24, 25].

On the other hand, Memè et al. [26] studied aligners exposed to coffee, tea, Coca-Cola, and ultraviolet rays for 24 and 48 h in laboratory conditions and found no alteration in the chemical properties. The same result was reported by Gerard Bradleyet al. [5] evaluating used and unused aligners.

Therefore, it can be said that there is a difference of opinion on this issue, which needs to be considered in further studies.

Finally, the hydrolysis process can affect the strength of the aligners. This is consistent with the findings of this study in the tensile strength section. As mentioned, after 10 days of intraoral use, the tensile strength of the aligners had decreased, although it was not statistically significant. This indicates that during the 10-day period of use, the aligner undergoes chemical changes at the molecular level, such as hydrolysis, but these changes are not substantial enough to produce detectable alterations in macroscopic physical or mechanical properties.

### Strengths and limitations

Unlike previous studies that were mostly conducted in vitro, this study was conducted in vivo, in which most of the properties of the aligners were examined at three levels: physical, mechanical, and chemical. However, as a limitation, it can be noted that tests such as color change were not performed because when this test is to be performed at the in vivo level, the samples must be grouped according to the type of food consumed.

### Suggestions for future research

Although changes in chemical composition are generally associated with alterations in mechanical and physical properties, our study did not reveal statistically significant differences in the evaluated mechanical parameters. Future research is warranted to investigate the underlying mechanisms and potential clinical relevance of such changes in orthodontic thermoplastic materials. It is suggested that such tests be performed in other studies where grouping is done for this purpose. It is also suggested that future studies examine the properties of the aligners in vivo in two different groups of aligners that have different materials, or at different times of use. Future studies with larger sample sizes are recommended to confirm these findings.

## Conclusion

After 10 days of intraoral use of aligners, the following occurs: no difference in water absorption is observed; water solubility increases, which is not statistically significant; elastic modulus increases, which causes the aligner to become harder, but this difference is not statistically significant; tensile strength decreases, which is not statistically significant; and the chemical properties of the aligner change. Therefore, no statistically significant effect was observed in the mechanical and physical properties of the aligners; however, their chemical properties can be altered with aging.

## Data Availability

The data generated or analyzed during this study are available from the corresponding author on logical request.

## Abbreviations

K-S: Kolmogorov–Smirnov
SD: Standard deviation
ATR-FTIR: Attenuated total reflectance-Fourier transform infrared
Wsl: Water solubility
Wsp: Water absorption
